# Supplementary material for: Safety and effectiveness of RBD-specific polyclonal equine F(ab´)2 fragments for the treatment of hospitalized patients with severe Covid-19 disease: A retrospective cohort study
Source: PLoS One. 2022 Sep 26;17(9):e0274796. doi: 10.1371/journal.pone.0274796 (PMC9512184; doi:10.1371/journal.pone.0274796)
Supplement: S1 File — (PDF) [file pone.0274796.s001.pdf]

Supporting information

Methods

Severity of illness categorization

The severity criteria for Covid-19 suggested by the National Institutes of Health (NIH) were used for the present study [1].

As such, we considered asymptomatic or presymptomatic Infection in those individuals who test positive for SARS-CoV-2 using a virologic test (i.e., a nucleic acid amplification test [NAAT] or an antigen test) but who have no symptoms that are consistent with COVID-19.

Mild illness in individuals who have any of the various signs and symptoms of COVID-19 (e.g., fever, cough, sore throat, malaise, headache, muscle pain, nausea, vomiting, diarrhea, loss of taste and smell) but who do not have shortness of breath, dyspnea, or abnormal chest imaging.

Moderate illness was considered for individuals who show evidence of lower respiratory disease during clinical assessment or imaging and who have an oxygen saturation (SpO2) ≥94% on room air at sea level. Severe illness was considered for individuals who have SpO2 <94% on room air at sea level, a ratio of arterial partial pressure of oxygen to fraction of inspired oxygen (PaO2/FiO2) <300 mm Hg, a respiratory rate >30 breaths/min, or lung infiltrates >50%.

And finally, Critical illness was considered for individuals who have respiratory failure, septic shock, and/or multiple organ dysfunction.

Data collection and verification process

Data collection process for EPIC cohort group

All data from EPIC patients in the cohort were retrieved from the prospective, structured registry designed within the frame of ANMAT´s Provision 4622/12 regarding authorization under special conditions. In addition, a review of the HCEH´s electronic medical records was performed for all selected patients in order to complete other data of interest. For the selection of the exposed patients a complete list of admissions included in the registry from the HCEH' between January 27th and April 17th, 2021, was reviewed. Patients with severe disease (defined as having respiratory rate of more than 30/min, or oxygen saturation <94% on room air at sea level, or a ratio of arterial partial pressure of oxygen to fraction of inspired oxygen (PaO2/FiO2) <300 mm Hg, or lung compromise of more than 50%) at the initiation of EPIC treatment within 24 hours of hospitalization were identified. Patients having moderate disease (including patients that received supplementary oxygen within 24 hours of hospitalization but with no documentation of tachypnea, oxygen desaturation or lung compromise above 50% through imaging) were excluded from this cohort group, as well as patients with mild and critical disease (including patients admitted to ICU, receiving mechanical ventilation or requiring inotropic drugs since hospital admission).

Review of the electronic medical records was started with the patients with earlier admission (January 27th, 2021) and moved forward toward the more recent admission dates until reaching the target sample of patients that had received at least one dose of EPIC. The medical data collection team retrieved the additional data of interest in a specific structured form developed for that purpose. After verification of completeness, a different team added the information to the electronic study database in an anonymized fashion.

Data collection process for the "Control" cohort group

For the selection of the "Control" patient group a complete list of all patients within 18- and 79-year-old admitted to the "HCEH" between November 25th, 2020 and January 21st, 2021 was obtained.

The medical data collection team thoroughly reviewed each electronic medical record in order to evaluate the selection criteria and completed the specific structured forms with the clinical data of the selected patients. This review was performed starting with the more recent admissions and moved backward until reaching the target sample complying the selection criteria. Again, a verification of completeness was performed before a different data team added this information to the electronic study database in an anonymized fashion.

Data validation process

A complete review and validation of all information included in the database was performed. An iterative process of generation of lists with all pending discrepancies and inconsistencies was implemented until final database closure. Site Principal Investigator signed electronically all final electronic case report forms that remained inalterable and protected from that moment onwards. Finally, a complete analysis of the data was carried out following the Statistical Analysis Plan.

Statistical Analysis Plan

Operationalization of variables

Age in years, BMI, PaO2/FiO2, Charlson Score and NEWS score were considered as continuous variables, while presence and type of comorbidities, prior use of convalescent plasma, diagnostic method, respiratory rate (≤20 or >20/min), requirement of supplementary oxygen and oxygen saturation (≤94 or >94%) were considered as categorical (dichotomous) variables. The thresholds for the categorization of numerical variables were defined by medical advice prior to the analysis of results.

Descriptive analysis

Categorical variables were presented as absolute and relative frequencies (percentage). Numerical variables were presented as mean and standard deviation or median and interquartile range whenever appropriate. Thresholds for the categorical variables were defined upon medical criteria and literature search prior to the process of data analysis.

All patient characteristics from the EPIC and Control groups were compared in order to detect potential confounders. Categorical variables were compared using the Chi square or Fisher's exact test and quantitative characteristics were compared using Student's T test or Mann Whitney's U test according with assumptions.

Although the study design itself may equalize overall EPIC and Control patient characteristics, since the authorization date for the use of EPIC at the HCEH is independent from the patient's characteristics, all association measurements were presented either as raw data and adjusted for inverse probability of treatment weighting (IPTW) and potential confounders, defining a doubly

robust method for estimation of the potential causal effect of the intervention of interest in comparison with the "non-exposed" cohort patients.

Given the observational nature of the study, all patient characteristics showing statistically significant differences were considered as potential confounders in addition to all variables identified "a priori" by researcher´s medical criteria.

Time to event without competing events

Taking into consideration the mortality events as right-censored events, there were no competing events for the primary outcome and its cumulative incidence was estimated using the Kaplan Meier method. Cumulative incidence curves according to time were presented at 28 days of follow up. Mortality cumulative incidence at days 14, 21 and 28 were estimated with 95% confidence intervals (CI95%). Median time to event and 25-75% percentiles were calculated. A comparison of survival curves between the two cohort groups was made with Cox-Mantel hypothesis test considering null hypothesis as survival curve overlap between EPIC and Control.

An univariate Cox proportional hazard regression model was used for estimation of the Hazard Ratio (HR) between cohort groups using death as result variable. In addition, an adjusted HR was obtained through the same regression model using IPTW and weighting by potential confounders (doubly robust approach). Both raw and adjusted HR were calculated with their respective CI95%.

Similar analysis was performed for hospital discharge as the interest variable.

Clinical ordinal scale analysis

An odds proportional ordinal regression model was used for the comparison of the distribution of the WHO-modified clinical ordinal scale between cohort groups at days 14, 21 and 28 of follow up. This model estimates a common OR for the difference between ordinal categories of the result variable. Proportional OR assumption was evaluated with Brant test (parallel regression assumption). Given potential difficulties in the interpretation of the proportional OR or possible violations to the proportional OR assumption, the ordinal scale similarity between cohort groups was analyzed with the Kruskal Wallis H test at days 14, 21 and 28 of follow-up.

Both raw OR as well as weighted by IPTW and potential confounders were presented with their respective CI95%.

Dichotomous categorical variables of safety and efficacy

Similar analysis was performed for all dichotomous secondary outcomes: proportion of patients discharged from hospital at days 14, 21 and 28, proportion of patients admitted to ICU, proportion of patients requiring mechanical ventilation and proportion of patients with any/serious adverse events.

Considering as null hypothesis an equal proportion of each secondary outcome between EPIC and “Control” groups, a Chi square or Fisher's exact test were used according to assumptions. Bivariate logistic regression model was used to estimate the raw OR of both cohort groups. Adjusted HR were obtained through the same regression model using IPTW and weighting by potential

confounders (doubly robust approach). Both raw and adjusted HR were calculated with their respective CI95%.

Time to event with competing events

Considering mortality as a competing event, the secondary outcomes time to hospital discharge, time to discharge from ICU and time to initiation of mechanical ventilation were analyzed using Kaplan Meier method with right censored data. Cumulative incidence curves according to time were presented at 28 days of follow up. Median time to event and 25-75% percentiles were calculated. A Fine and Gray bivariate regression model considering death as a competing event was used for estimation of sub–Hazard Ratios (sHR) for each cohort group, using referred secondary outcomes as result variables. Similar Fine and Gray multivariate regression model was used weighted by IPTW and adjusted for potential confounders for estimation of the adjusted sHR. Both raw and adjusted sHR were presented with respective CI95%.

Adjustment for potential confounders - Causal estimators

Weighting by the inverse probability of receiving treatment (IPTW) and furtherly by potential confounders was implemented for adjustment by unbalanced confounders between EPIC and “Control” groups, in a doubly robust approach. In this way, association measures weighted by IPTW and adjusted for potential confounders correspond to the average causal effect in the population (Average Treatment Effect in the population, ATE).

A multivariate logistic regression model was used for calculation of the propensity score using exposition to EPIC as dichotomous response variable. All other identified potential causes of exposition or death were included as explanatory variables. In addition, all unbalanced variables and those considered as potential predictors of EPIC use or reflecting changes in diagnosis, staging, concomitant treatment or support measures between cohort groups were included as explanatory variables.

We estimated the propensity score (PS) of EPIC exposure using a logistic regression model with EPIC exposure as dependent variable and the following potential predictors of treatment: gender at birth, age, clinical parameters at cohort admission (respiratory rate, heart rate, body temperature, oxygen saturation), requirement of supplementary oxygen or non-invasive ventilation, Charlson´s Score, National Early Warning Score (NEWS), time from symptoms onset, prior use of angiotensin converting enzyme inhibitors, non-steroidal antiinflammatory agents, corticosteroids, heparin, immunosuppressors, ivermectin or statins; presence and number of comorbidities: obesity, cardiovascular disease, stroke, hemiplegia, arterial hypertension, chronic lung disease, chronic renal disease, dementia, peptic ulcer, diabetes with or without target organ damage, solid organ tumor or leukemia. With this propensity score we calculated the stabilized IPTW. The weights were truncated at percentile 1% and 99% in order to avoid extreme figures. The distribution overlap between EPIC and Control groups were verified using histogram figures. Overall variable balance after IPTW adjustment is shown in S1 Fig.

A null regression model with the same response variable but without explanatory variables was used for estimation of the marginal probability (MP) of the exposition to EPIC. With the propensity score and marginal probability, the individual weighting was calculated for each participant as the

stabilized inverse probability of treatment. This weighting is defined as PS/MP for the "EPIC" patient group and (1-PS)/(1-MP) for the "Control" patient group.

Multivariate regression models weighted for IPTW and adjusted for potential confounders were used for the estimation of ATE with the doubly robust approach. Standardized bias (standardized differences) was compared before and after applying IPTW. All standardized biases below 0,2 after the use of IPTW were considered appropriate. All data were presented using Love plots with the STATA command defined by pbalchk user version 3.0.0 generated by Lunt¨ [2, 3].

Efficacy subgroup analysis

A subgroup analysis was predefined for efficacy. Subgroup analysis results were presented as the P-value of the interaction test and the OR calculated for each subgroup. Based upon clinical interest, the following subgroups were pre-specified: gender at birth, age category groups (less than 65, or between 65 and 79 years old), time from symptoms initiation (less or more than 3 days, less or more than 5 days or between 5 and 10 days), obesity, presence and number of main comorbidities (immunosuppression, diabetes, arterial hypertension, cardiovascular disease) and obesity.

All tests were two-sided, and a P value < 0.05 was considered statistically significant. All statistical analysis was performed using STATA statistical software version 15.1 MP - Parallel Edition (Copyright 1985-2017 StataCorp LLC - StataCorp. 4905 Lakeway Drive, College Station, Texas 77845 USA).

Results

Population characteristics

A complete description of patients´ comorbidities at cohort entry is shown in S1 Table.

Secondary analyses

Patient's discharge at day 14 was significantly greater in the EPIC group than in the Control group -280 (79,9%) vs 281 (63%) OR 1.46 (95% CI 1.07 to 1.98), P=0.016 for IPTW adjustment. There were no differences between cohort groups in the rest of the secondary outcomes evaluated.

Other than mortality and WHO-modified ordinal clinical scale results, the complete results of secondary outcomes are provided in S2 Table.

Sensitivity analyses

Sensitivity analysis for mortality performed to subjects that received two complete doses of EPIC in comparison with Control group showed a significantly greater effect of the intervention (OR 0.58 [95% CI 0,39 to 0.85] for IPTW adjustment and OR 0.57 [95% CI 0.37 to 0.86] for the doubly robust approach).

Sensitivity analysis comparing patients on the EPIC group with patients in the Control group that received convalescent plasma showed that the effect remained significant in favor of the

intervention (OR 0.62 [95% CI 0.42 to 0.93] for IPTW and OR 0.61 [95% CI 0.39 to 0.94]) for doubly robust analysis. Complete results of the sensitivity analyses are shown in S3 Table.

We performed an additional sensitivity analysis with the respiratory rate and oxygen saturation variables considered as continuous. In such case the OR for the primary outcome was 0.75 (95% CI 0.51 to 1.10) P=0.142 and the HR 0.78 (95% CI 0.55 to 1.10) P=0.160 for the IPTW adjustment, and OR 0.72 (95% CI 0.47 to 1.09) P=0.122 and HR 0.77 (95% CI 0.54 to 1.09) P=0.136 for the doubly robust approach.

Safety

No significant differences were found in the breakdown of adverse events according to systems and organs. A detailed description of the AEs between cohort groups can be found in S4 Table.

References

1. Available at: https://www.covid**19treatmentguidelines.nih.gov/overview/clinical-spectrum/** (accessed March 2022)
2. Available at: https://www.hcp.med.harvard.edu/sites/default/files/Methods%20for%20Constructing%20and%20Assessing%20Propensity%20Scores.pdf
3. [No title]. [cited 3 Apr 2021]. Available at: http://personalpages.manchester.ac.uk/staff/mark.lunt/pbalchk.ado

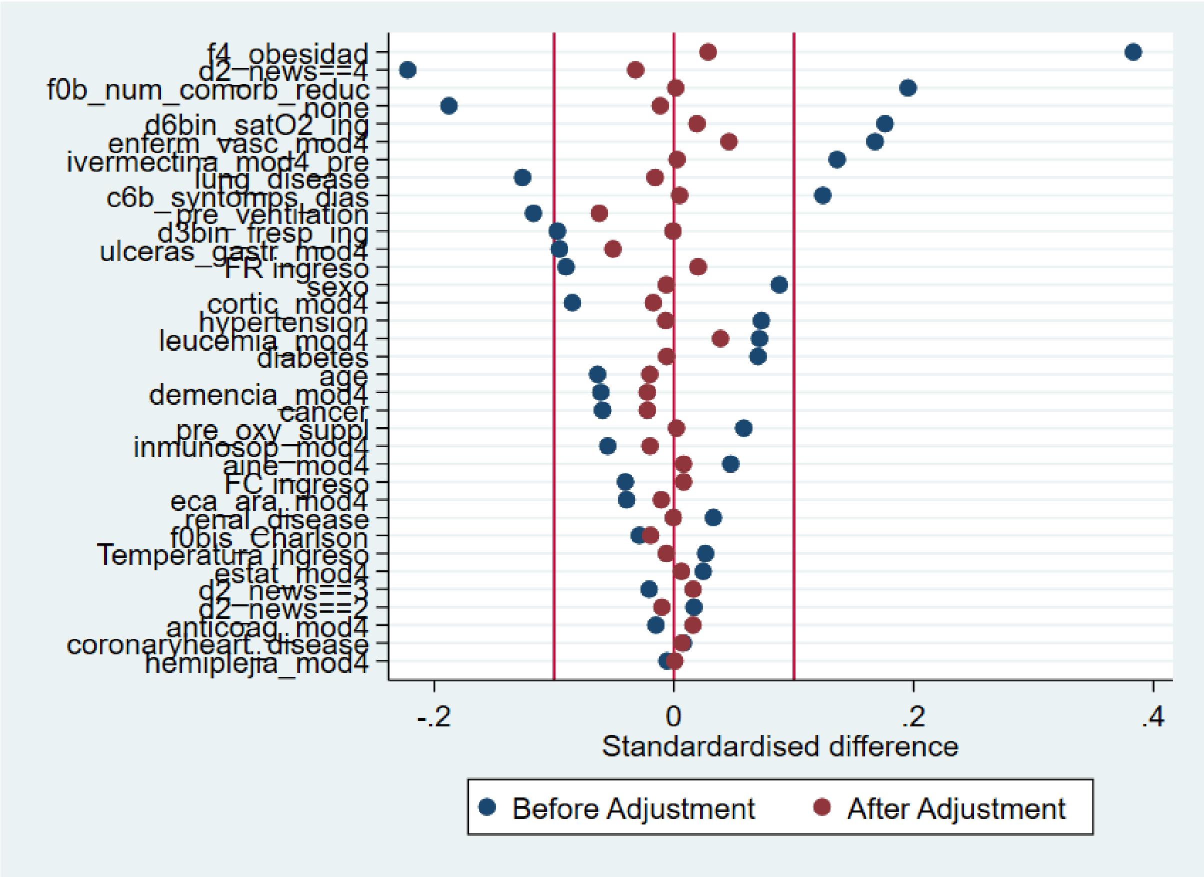

S1 Fig. Balance assessment of study variables before and after IPTW adjustment.

	EPIC (N=395)	Control (N=446)	P value
**Coexisting conditions**			
**Charlson score**	0 (0 - 2)	0 (0 - 2)	0.9982†
**Number of comorbidities#**	2 (1 - 3)	1 (1 - 3)	0.0003†
**None**	9.1 (36)	15.0 (67)	0.009
**Hypertension**	57.7 (228)	54,3 (242)	0.313
**Diabetes**	28.1 (111)	24.7 (110)	0.258
**Obesity**	59.5 (235)	40.6 (181)	<0.001
**Cancer**	2.0 (8)	2.9 (13)	0.409
Tumor without metastasis	1.8 (7)	2.2 (10)	0.807‡
Tumor with metastasis	0 (0)	0.7 (3)	0.252‡
Leukemia	0.3 (1)	0 (0)	0.470‡
Lymphoma o Myeloma multiple	0 (0)	0 (0)	
**Lung disease**	7.1 (28)	10.5 (47)	0.080
**Liver disease**	0.8 (3)	0.7 (3)	1.000‡
**Renal disease**	3.04 (12)	2.7 (12)	0.763
**Coronary and cardiovascular disease**	6.3 (25)	6.3 (28)	0.976

**Stroke / Peripheral vascular disease**	4.8 (19)	2.0 (9)	0.024
**Dementia**	1.3 (5)	2.0 (9)	0.395
**Connective tissue disease**	1.5 (6)	1.4 (6)	0.832
**Gastric ulcer**	0 (0)	0.5 (2)	0.501‡
**Hemiplegia**	1.5 (6)	1.6 (7)	0.953
**AIDS**	0 (0)	0.2 (1)	1.000‡
**Others** (including hypothyroidism, dislipidemia, gastrointestinal disease, drug allergies and rheumatic disease).	37.2 (147)	28.3 (126)	0.006

S1 Table. Cohort patient´s comorbidities at entry.

Outcomes	EPIC (N=395)	Control (N=446)	Estimator	Crude	IPTW†	Doubly robust adjustment‡
Secondary outcomes						
Patients with hospital discharge at day 28	78.5 (310)	75.6 (337)	OR	1.17 (95%CI 0.85 - 1.61) p 0.344	1.17 (95%CI 0.83 - 1.64) p 0.374	1.16 (95%CI 0.80 - 1.68) p 0.445
Patients with hospital discharge at day 21	76.2 (301)	72.9 (325)	OR	1.18 (95%CI 0.87 - 1.62) p 0.293	1.17 (95%CI 0.84 - 1.62) p 0.356	1.16 (95%CI 0.81 - 1.66) p 0.428

Patients with hospital discharge at day 14	70.9 (280)	63 (281)	OR	1.42<br>(95%CI 1.06 - 1.90)<br>p 0.018	1.46<br>(95%CI 1.07 - 1.98)<br>p 0.016	1.49<br>(95%CI 1.07 - 2.09)<br>p 0.018
Time until discharge (days)#	9 (6-15)	10 (6-17)	sHR	1.08<br>(95%CI 0.93 - 1.25)<br>p 0.298	1.07<br>(95%CI 0.92 - 1.25)<br>p 0.349	1.07<br>(95%CI 0.92 - 1.25)<br>p 0.402
Patients requiring ICU admission	20 (79)	23.8 (106)	OR	0.8<br>(95%CI 0.58 - 1.11)<br>p 0.189	0.73<br>(95%CI 0.51 - 1.02)<br>p 0.069	0.71<br>(95%CI 0.49 - 1.03)<br>p 0.072
Time since admission until discharge from ICU (days)#	13 (11 - 23)	13.5 (7 - 18)	sHR	0.57<br>(95%CI 0.23 - 1.37)<br>p 0.207	0.47<br>(95%CI 0.19 - 1.15)<br>p 0.1	0.41<br>(95%CI 0.16 - 1.01)<br>p 0.054
Patients requiring invasive mechanical ventilation	18.7 (74)	20.6 (92)	OR	0.88<br>(95%CI 0.63 - 1.25)<br>p 0.491	0.82<br>(95%CI 0.57 - 1.17)<br>p 0.270	0.82<br>(95%CI 0.55 - 1.20)<br>p 0.299
Time since admission until MV requirement (days)#	ND	ND	sHR	0.93<br>(95%CI 0.68 - 1.26)<br>p 0.642	0.86<br>(95%CI 0.71 - 1.38)<br>p 0.366	0.87<br>(95%CI 0.63 - 1.21)<br>p 0.420

ND: could not be determined

S2 Table. Secondary outcomes

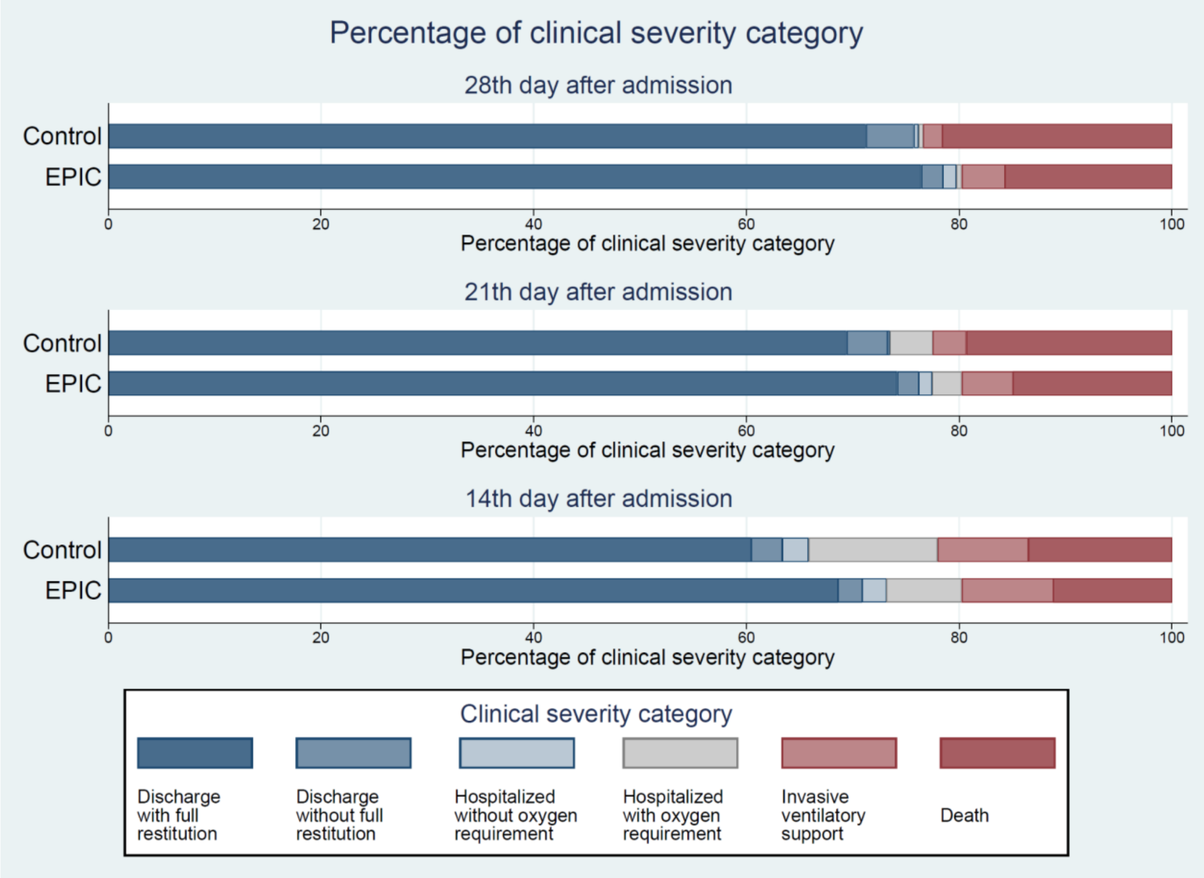

S2 Fig. WHO 6-points ordinal clinical scale measured at days 14, 21 and 28

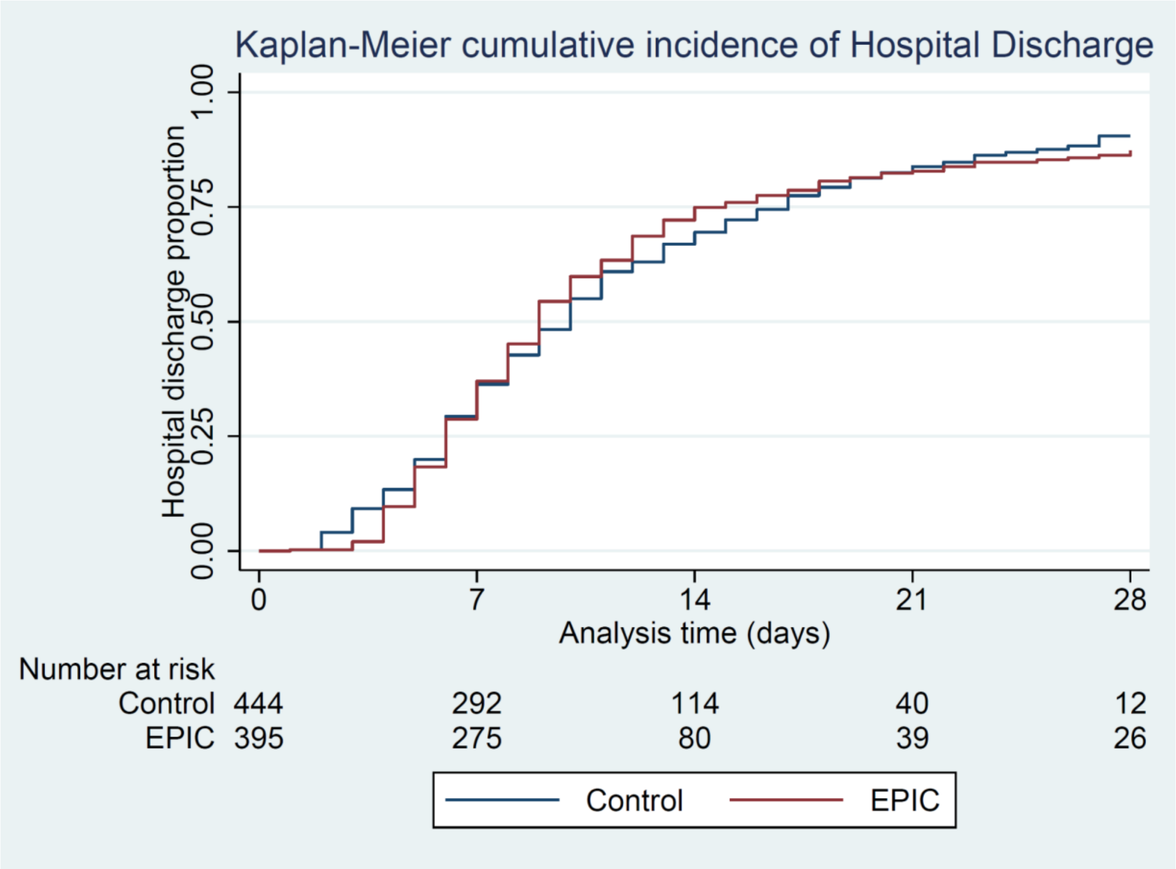

S3 Fig. Incidence in hospital discharge (days)

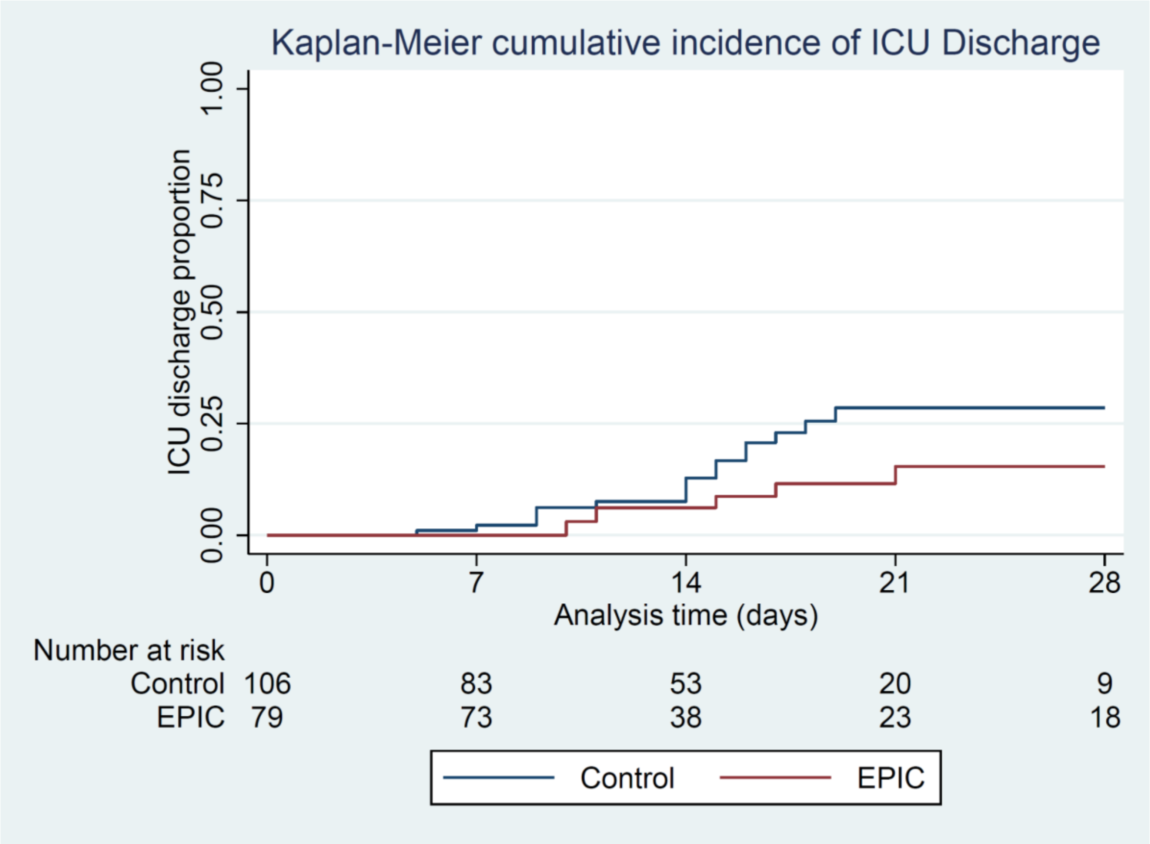

S4 Fig. Time since admission until discharge from ICU (days)

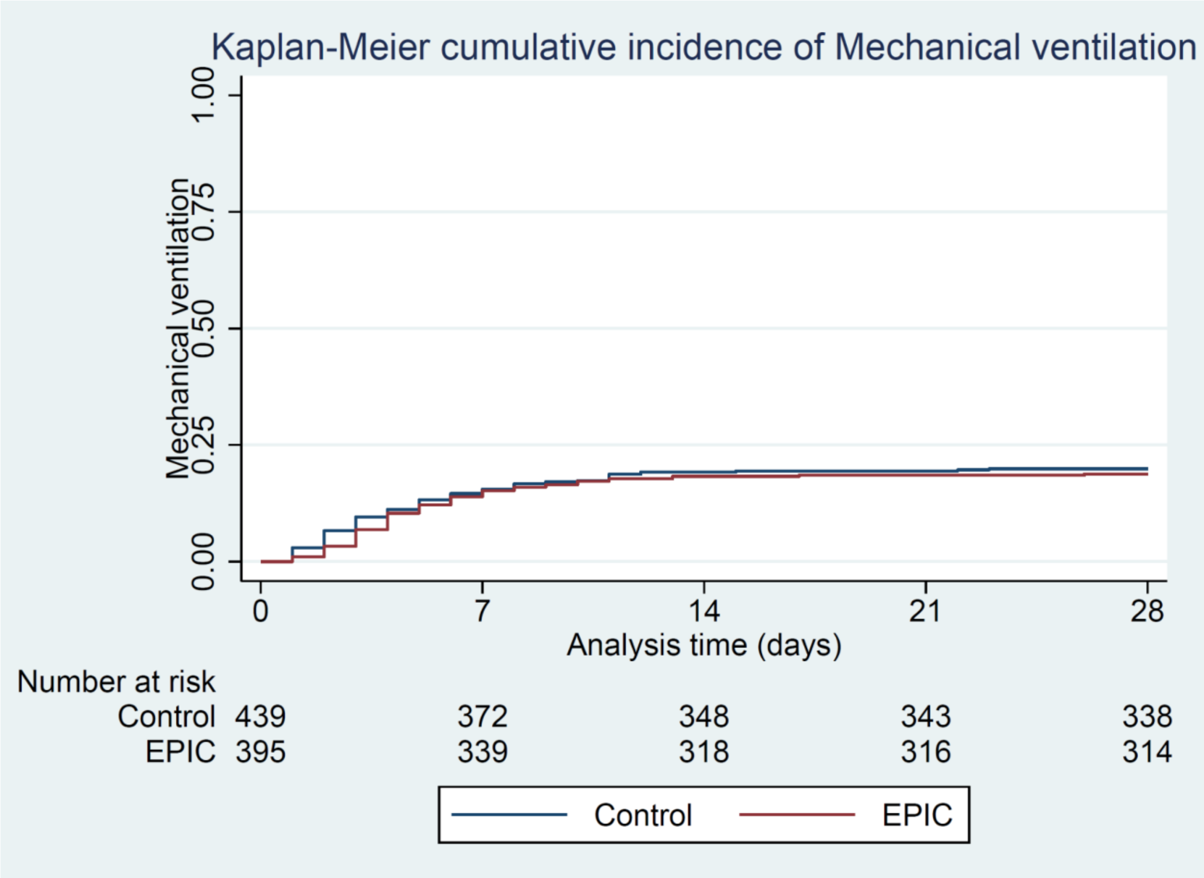

S5 Fig. Time since admission until MV requirement (days)

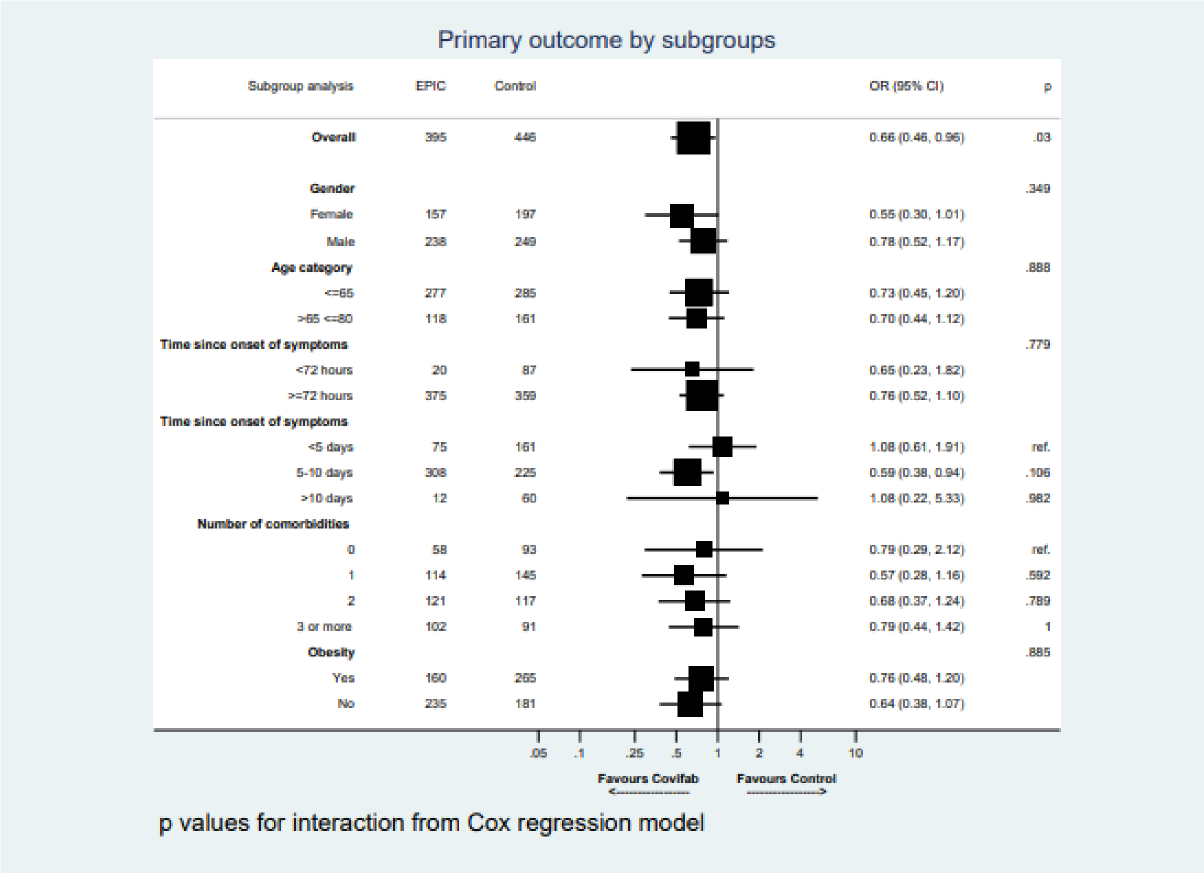

S6 Fig. Subgroup analysis

**Primary outcome- Sensitivity analysis** in patients with complete EPIC treatment						
**Outcomes**	**EPIC (N=379)**	**Control (N=446)**	**Estimator**	**Crude**	**IPTW†**	**Doubly robust adjustment‡**
Overall mortality at day 28 since hospital admission	14 (53)	21.5 (96)	OR	0.59 (95%CI 0.41 - 0.86) p 0.005	0.58 (95%CI 0.39 - 0.85) p 0.005	0.57 (95%CI 0.37 - 0.86) p 0.008
			HR	0.63 (95%CI 0.45 - 0.88) p 0.007	0.61 (95%CI 0.43 - 0.87) p 0.007	0.63 (95%CI 0.44 - 0.91) p 0.013

**Primary outcome- Sensitivity analysis** Patients with Convalescent plasma versus EPIC						
**Outcomes**	**EPIC (N=395)**	**Control with Convalescent plasma (N=317)**	**Estimator**	**Crude**	**IPTW†**	**Doubly robust adjustment‡**
Overall mortality at day 28 since hospital admission	15.7 (62)	22.7 (72)	OR	0.63 (95%CI 0.43 - 0.92) p 0.018	0.62 (95%CI 0.42 - 0.93) p 0.019	0.61 (95%CI 0.39 - 0.94) p 0.025
			HR	0.67 (95%CI 0.48 - 0.95) p 0.023	0.66 (95%CI 0.46 - 0.95) p 0.023	0.68 (95%CI 0.47 - 1) p 0.048

S3 Table. Sensitivity analyses

	EPIC (N=395)	Control (N=446)
**Any patient with an adverse event**	24.8 (98)	27.1 (121)
**Total of adverse events**	145	168
**System organ classification**		
**Blood and lymphatic system disorders**	0	0
**Cardiac disorders**	0.2 (1)	0
**Ear and labyrinth disorders**	0	0
**Endocrine disorders**	0	0
**Eye disorder**	0	0
**Gastrointestinal disorders**	0.2 (1)	0.2 (1)
**General disorders and administration site conditions**	9.6 (38)	11.1 (44)
**Hepatobiliary disorders**	0	0
**Immune system disorders**	0	0
**Infections and infestations**	4.6 (18)	0.7 (3)
**Injury, poisoning and procedural complications**	0	0
**Investigations for laboratory test results**	0	0

	EPIC (N=395)	Control (N=446)
**Any patient with an adverse event**	24.8 (98)	27.1 (121)
**Total of adverse events**	145	168
**Metabolism and nutrition disorders**	0	0
**Musculoskeletal and connective tissue disorders**	0	0
**Neoplasms benign, malignant and unspecified (incl cysts and polyps)**	0	0
**Nervous system disorders**	0.2 (1)	0
**Pregnancy, puerperium and perinatal conditions**	0	0
**Psychiatric disorders**	0	0
**Renal and urinary disorders**	0.2 (1)	0
**Reproductive system and breast disorders**	0	0
**Respiratory, thoracic and mediastinal disorders**	20.3 (80)	26.9 (120)
**Skin and subcutaneous tissue disorders**	10.1 (4)	0
**Surgical and medical procedures**	0	0
**Vascular disorders**	0.2 (1)	0
**Non-Covid death**	0	0

S4 Table. Breakdown of Adverse Events between groups
